# Stress-Induced Cardiomyopathy Secondary to COVID-19

**DOI:** 10.1155/2020/8842150

**Published:** 2020-08-24

**Authors:** Aneesh Bapat, Abhishek Maan, E. Kevin Heist

**Affiliations:** Cardiac Arrhythmia Service, Massachusetts General Hospital, Boston, MA 02114, USA

## Abstract

A 67-year-old female with prior medical history of HTN and asthma presented with acute-onset dyspnea and nausea for 4 days prior to admission. Upon initial encounter in the emergency room, she was found to have findings of abnormal pulmonary infiltrates and consequent workup revealed COVID-19. During further hospital course, the patient developed abnormal EKG and echocardiographic findings consistent with stress-induced cardiomyopathy.

## 1. Introduction

As the world deals with the ongoing SARS-CoV2 pandemic, there is growing recognition of its cardiovascular effects, which include myocarditis, arrhythmias, and thromboemboli. Given the degree to which the pandemic has affected daily life, it is associated with significant social and emotional stress in addition to its health-related effects. Herein, we describe a case of stress-related cardiomyopathy—first recognized via marked repolarization abnormalities on electrocardiogram—in a patient with SARS-CoV2 in the absence of ongoing competing cardiovascular issues. We discuss the typical electrocardiographic findings related to stress-induced cardiomyopathy and survey the literature for mechanistic insights. As the ongoing pandemic continues to take its course, it will be important to remain vigilant about the electrical findings of this entity, which are accompanied by an increased risk of arrhythmia.

## 2. Case Presentation

### 2.1. History of Present Illness

A 67-year-old female presented to the ER with approximately 4 days of exertional dyspnea. These symptoms were accompanied with significant nausea. Despite the use of rescue inhalers, her symptoms persisted and she sought further medical attention in the ER. On EMS arrival, she was found to have an oxygen saturation < 90% on room air and required oxygen supplementation with a nonrebreather.

### 2.2. Past Medical History

The patient's relevant past medical history was remarkable for asthma, HTN, and DM: all of these were well controlled. She did not have any prior structural heart disease history; however, a prior echocardiogram (which was done for evaluation of syncope) in 2006 had shown left ventricular ejection fraction of 65% without any regional wall motion abnormalities. In addition, a noncontrast chest CT done within a year of this admission incidentally showed mild to moderate coronary and aortic arch calcification.

### 2.3. Emergency Room Evaluation

**Figure 1:**
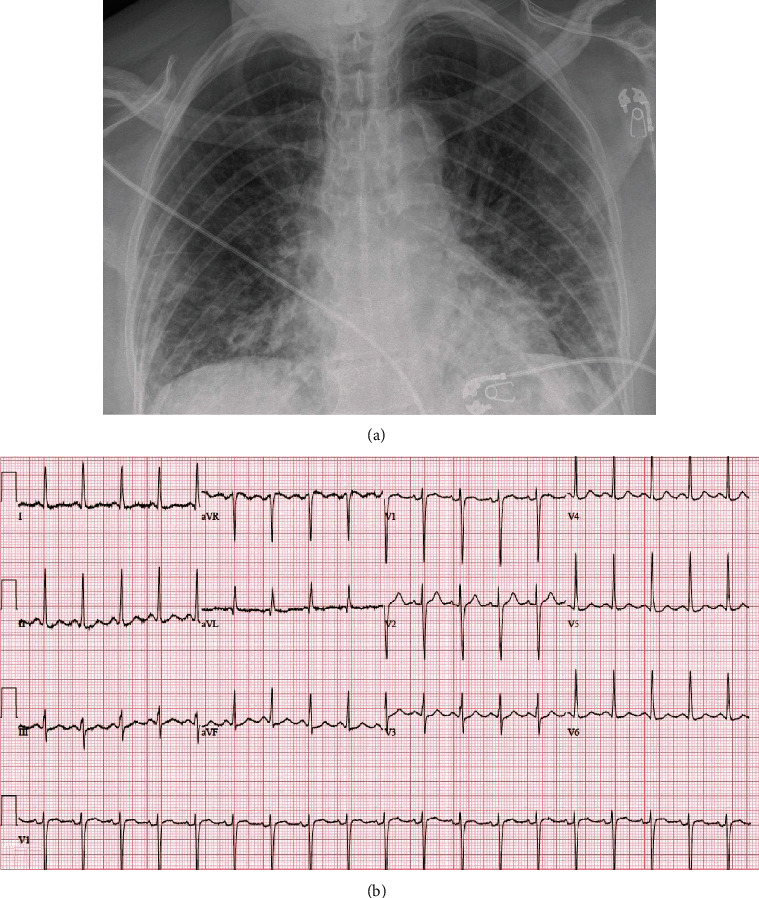
(a) Chest X-ray at time of presentation, consistent with the diagnosis of SARS-CoV2. (b) EKG at time of presentation.

On arrival to the ER, the patient was found to be tachycardic with a HR of 118 bpm (Figure 1(b)) and required 2-4 liters of supplemental oxygen. Chest XR revealed “bilateral predominantly peripherally distributed patchy opacities” (Figure 1(a)). Her initial laboratory investigation was remarkable for elevations in LDH, CK, and inflammatory markers, but with a normal high-sensitivity troponin T (Table 1). The polymerase chain reaction test for SARS-CoV2 was detected to be positive.

### 2.4. Initial Hospital Course

The patient was admitted to the inpatient non-ICU unit and was primarily managed with supportive care. She required 2-3 l/min of O_2_ during the day and 4-6 l/min of O_2_ overnight along with nocturnal proning. She did not require treatment with any investigational pharmacotherapy including hydroxychloroquine, remdesivir, or biologic monoclonal antibodies.

### 2.5. Clinical Decompensation and ICU Course

On the fourth day of her hospitalization, the patient ambulated to the restroom and on return had a desaturation to <85% oxygen. She required a rapid uptitration of supplemental oxygen via facemask, so she was transferred to the ICU for closer monitoring and consideration of mechanical ventilation. Given the ongoing increase in her supplemental oxygen requirements, the patient was nonemergently intubated to avoid further decompensation. As a result of the sedation required for intubation, the patient developed mild hypotension requiring a low dose of norepinephrine support. Over the course of the day, her pressor requirement remained unchanged, and her respiratory status was stable on minimal ventilation settings with a PaO_2_/FiO_2_ ratio of >150.

**Figure 2:**
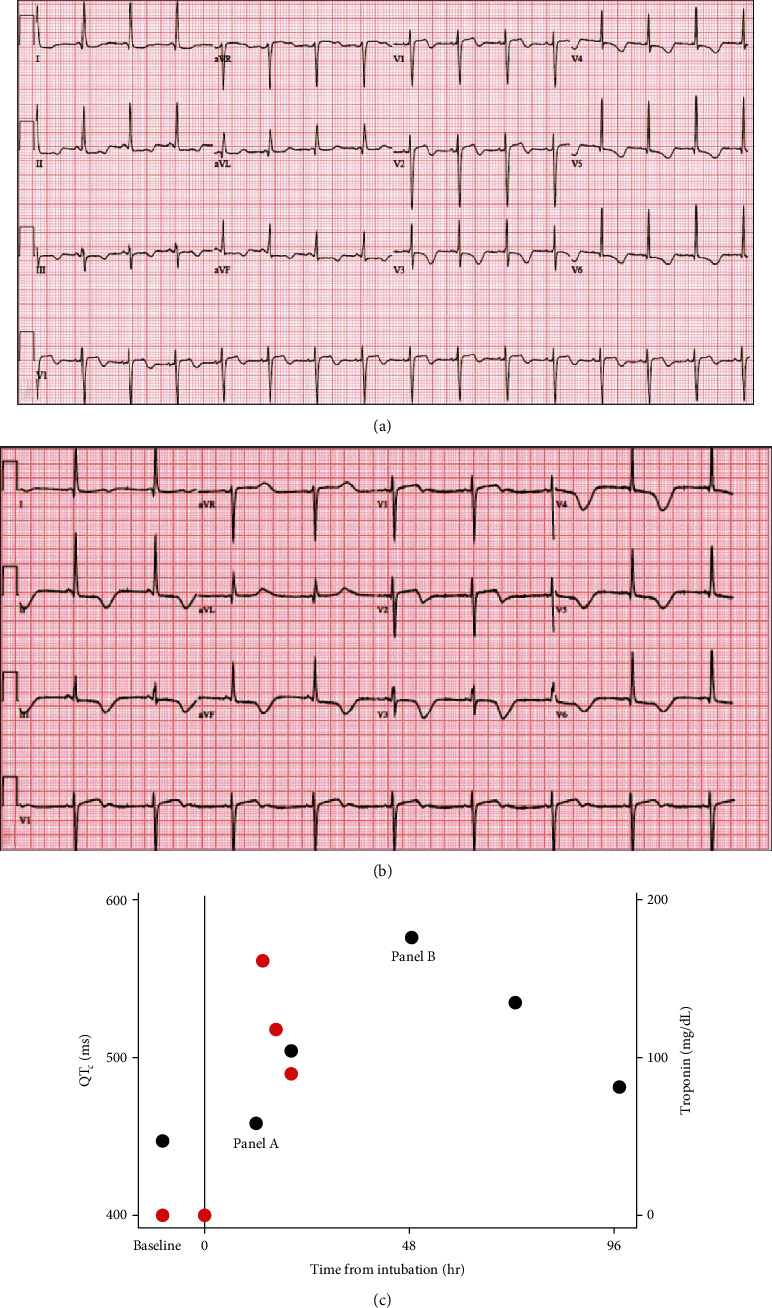
(a) EKG done approximately 12 hours after respiratory decompensation requiring intubation showing ST elevations and biphasic T waves. (b) EKG done approximately 48 hours after intubation showing marked QT prolongation. (c) Time course of QTc and hsTnT values after intubation.

**Figure 3:**
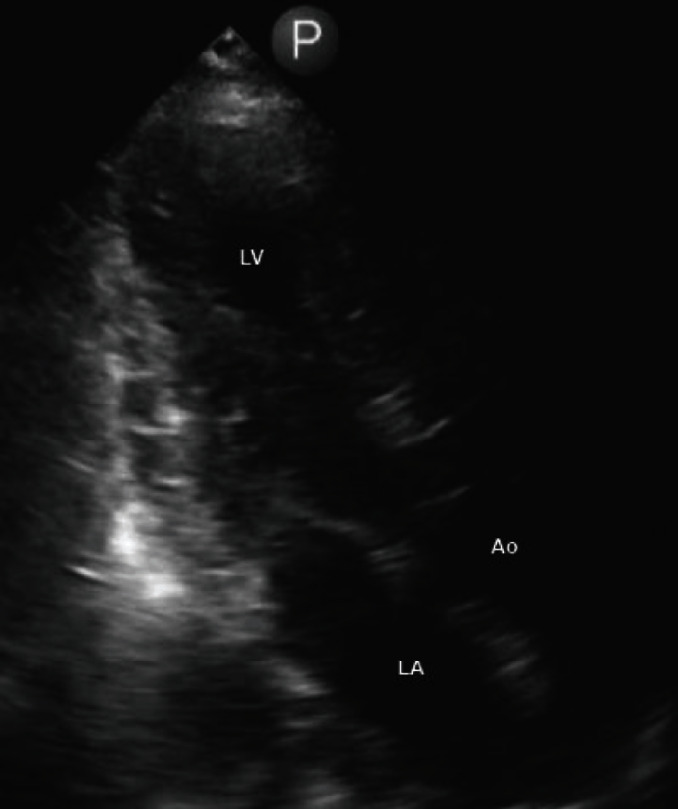
Image of the apical view of transthoracic echocardiogram showing apical hypokinesis.

On the night of her respiratory decompensation and transfer to the ICU, a routine EKG was done and was remarkable for new repolarization abnormalities (Figure 2(a)). At that time, there was no clinical decompensation or change in her ventilatory or pressor support requirements. Over the following 48 hours, serial EKGs (Figures 2(b)–2(c)) showed continued persistence of T wave inversions and progressive prolongation in the QT interval. High-sensitivity troponin levels were checked at the time of the patient's initial respiratory decompensation and then again in the context of the EKG finding; there was biomarker evidence of cardiac injury which quickly resolved (Figure 2(c)). A TTE done approximately 24 hours after the patient's respiratory decompensation revealed preserved left ventricular ejection fraction of 61% but with new apical hypokinesis. (Figure 3, Supplemental Video #1).

### 2.6. Differential Diagnosis

We suspect that the patient's clinical decompensation was secondary to respiratory decompensation which has been well-reported in light of the current COVID-19 pandemic. Additionally, we suspect that the subsequent electrical and structural cardiac abnormalities resulted from a stress-induced cardiomyopathy. However, the differential diagnosis includes coronary ischemia, and an ischemic evaluation is warranted when clinically relevant. The remainder of the differential diagnosis includes a myocarditis perhaps related to viral infection, a drug-related QT prolongation, and repolarization changes related to metabolic disarray; there is no evidence to support any of these diagnoses.

### 2.7. Follow-Up

Over the subsequent 2 weeks, the patient was gradually weaned from ventilator and pressor support. She was managed conservatively without any specific antiviral medications including hydroxychloroquine. Given the concern for LV dysfunction, she was started on metoprolol, and this was continued at discharge. Her HTN treatment regimen included lisinopril, which was continued. Her EKG abnormalities gradually resolved, with progressive normalization of her QT interval. She was eventually discharged to rehabilitation and will follow up with a plan for an outpatient reassessment of her LV function.

## 3. Discussion

We describe a case of a young patient who had initially presented with acute respiratory illness secondary to COVID-19 which rapidly progressed to hypoxic respiratory failure and required mechanical ventilatory support on day 8 after symptom onset. Her course has been complicated by biomarker evidence of myocardial injury, marked repolarization abnormalities, and TTE showing apical hypokinesis with sparing of basal segment along with overall preserved LV function. Overall, these data are supportive of a diagnosis of a stress-induced cardiomyopathy resulting from COVID-19 infection and correlated temporally to the timing of intubation.

In general, this is considered a diagnosis of exclusion, and in this case, the most glaring differential diagnosis is a critical coronary lesion affecting her apex. Unfortunately, given the patient's continued respiratory recovery, an ischemic evaluation has not yet been pursued, but this would be appropriate at time of follow-up. However, the relatively mild elevation in hs-troponin in her case seemed to correlate very well with the timing of intubation. Furthermore, the troponin elevation quickly resolved while the EKG changes continued to be persistent (Figure 2(c)) in the context of typical wall motion abnormalities of Takotsubo cardiomyopathy.

COVID-19 has led to a rapid increase in hospital admissions and mortality across the globe. Based on the initial publications, the patients with COVID-19 infection presented with myriad of symptoms ranging from fever, upper respiratory infection, and acute kidney injury. Additionally, a significant proportion of patients have also been reported to have myocarditis and acute cardiac injury. Currently published reports have reported a correlation between acute cardiac injuries with an increased risk of overall mortality.

Stress cardiomyopathy, also known as Takotsubo cardiomyopathy (TCM), was first described in 1991 and initially thought to be the result of multivessel coronary spasm in the absence of fixed epicardial coronary disease [1]. Cardiovascular findings included a typical echocardiographic pattern of apical hypokinesis with basal hyperkinesis. This cardiomyopathy—later described as “broken heart syndrome”—was found to be associated with sudden emotional stress and elevated sympathetic stimulation [2]. Triggers can be emotional or physical stressors, and in some cases, the cardiomyopathy has also been reported in the context of ongoing treatment of another critical illness. Our patient presented with SARS-CoV2 infection and developed rapid-onset respiratory distress requiring intubation and so was certainly exposed to sufficient stressors which would contribute to the development of TCM. Short-term consequences may include fulminant heart failure and cardiogenic shock, outflow tract obstruction, arrhythmias, and systemic thromboembolism. Management is generally supportive with special care taken to be vigilant for and aggressively treat the complications. Recovery of LV function is gradual but generally occurs over the course of weeks to months [3].

The EKG changes seen in this patient are typical for Takotsubo cardiomyopathy, which tends to primarily affect ventricular repolarization [2]. An early description of the temporal evolution of EKG changes in patients with TCM was consistent with that in the patient presented here: initial ST-segment elevations as well as inverted T waves in V3-V6 with progressive deepening of the TWI over the subsequent 2-3 days associated with a prolongation in QTc [4]. Given the overlap in presentation as compared to an acute myocardial infarction, there are a number of EKG-based criteria that have been validated to differentiate TCM and acute myocardial infarction [5]. Although these changes in repolarization generally resolve in the long term [4], they carry an increased risk of potentially life-threatening ventricular arrhythmias [6].

The mechanisms underlying TCM have been elusive, but a catecholamine surge seems to be the unifying driver with consequent endothelial dysfunction—including vasospasm and SCAD—and inflammation [7–9]. Similarly, the etiology of the EKG changes and altered ventricular repolarization is yet to be defined and any explanation is speculative based on analogous model systems. Animal models of TCM generally involve an acute injection of isoproterenol in rats, which results in a transient apical akinesis associated with evidence of calcium- (Ca^++^-) overload-induced contractile dysfunction as well as increased diastolic Ca^++^ leak from the sarcoplasmic reticulum [10, 11]. Although the latter may certainly be arrhythmogenic, it would not explain the QT prolongation. The effect of sympathetic tone on ventricular repolarization is more complicated, as catecholamines such as epinephrine increase the flux through both repolarizing outward K^+^ currents and depolarizing inward Ca^++^ currents; the net effects determine the overall effect on QT interval [12]. Interestingly, theoretical models as well as studies in isolated myocytes have suggested that the acute effects of catecholamines may preferentially affect calcium currents, leading to transient APD prolongation and early afterdepolarizations. These effects reflect differential kinetics, which only occur with acute exposure to catecholamines, and resolve in the steady state [13]. The dynamic effects described certainly mimic the clinical observation of transient QT prolongation seen with stress-induced cardiomyopathy.

Although the primary insults from SARS-CoV2 infection are respiratory, there has been a wide variety of diverse multiorgan manifestations. There are case reports of TCM in the context of a pericardial effusion [14] as well as a reverse TCM in the context of myocarditis [15]. Here, we present a case of TCM in the absence of any other cardiac insults. However, the SARS-CoV2 pandemic presents a myriad of potential triggers for TCM which include not only acute illness but also the social and emotional factors. The profound EKG findings are of special importance in this context. Although the use of hydroxychloroquine has fallen out of favor, treatment with azithromycin remains in the arsenal for most patients, both of which remain to be associated with the risk of QT prolongation. Additionally, early observations made during the pandemic have also suggested an increased risk of arrhythmia and sudden cardiac death associated with the virus. As the SARS-CoV2 pandemic continues to escalate, it would be prudent to remain vigilant towards the structural and electrical adverse effects which occur with TCM in the setting of acute illness.

## 4. Conclusions

We report a case of TCM with typical electrical and structural manifestations in the context of acute respiratory illness secondary to COVID-19 infection.

## Figures and Tables

**Table 1:** Summary of initial laboratory investigations done at the time of admission.

Laboratory parameter	Observed values	Reference values
C-reactive protein (CRP)	138.4 mg/l	<8.0 mg/l
Serum ferritin	328 *μ*g/l	10-100 *μ*g/l
Serum creatinine	0.76 mg/dl	0.60-1.50 mg/l
Lactate dehydrogenase (LDH)	318 U	110-210 U/l
NT-proBNP	474 ng/l	0-900 ng/l
hs-troponin	10 ng/l	0-9 ng/l

## Abbreviations

HTN: Hypertension
COVID-19: Coronavirus disease 19
SARS-CoV2: Severe acute respiratory syndrome coronavirus 2
ER: Emergency room
EMS: Emergency medical services
DM: Diabetes mellitus
HR: Heart rate
CT: Computed tomography
LDH: Lactate dehydrogenase
CK: Creatine kinase
XR: X-ray
ICU: Intensive care unit
EKG: Electrocardiogram
TTE: Transthoracic echocardiogram
LV: Left ventricle
APD: Action potential duration.

## Supplementary Materials

Supplementary materials Supplemental Video #1: a 3-chamber transthoracic echocardiogram showing apical hypokinesis with preserved basal LV function.
